# Chronic critical illness and post-intensive care syndrome: from pathophysiology to clinical challenges

**DOI:** 10.1186/s13613-022-01038-0

**Published:** 2022-07-02

**Authors:** Guillaume Voiriot, Mehdi Oualha, Alexandre Pierre, Charlotte Salmon-Gandonnière, Alexandre Gaudet, Youenn Jouan, Hatem Kallel, Peter Radermacher, Dominique Vodovar, Benjamine Sarton, Laure Stiel, Nicolas Bréchot, Sébastien Préau, Jérémie Joffre, la CRT de la SRLF

**Affiliations:** 1 Service de Médecine Intensive Réanimation, Hôpital Tenon, Sorbonne Université, Assistance Publique - Hôpitaux de Paris, Paris, France; 2 Pediatric Intensive Care Unit, Necker Hospital, APHP, Centre - Paris University, Paris, France; 3 Institut Pasteur de Lille, U1167 - RID-AGE - Facteurs de Risque et Déterminants Moléculaires des Maladies Liées au Vieillissement, University Lille, Inserm, CHU Lille, 59000 Lille, France; 4 Department of Intensive Care Medicine, Critical Care Center, CHU Lille, 59000 Lille, France; 5 Faculté de Médecine de Tours, Centre d’Etudes des Pathologies Respiratoires, INSERM U1100, University Lille, Tours, France; 6 Service de Médecine Intensive Réanimation, CHRU de Tours, Réseau CRICS-TRIGGERSEP F-CRIN Research Network, Tours, France; 7 Institut Pasteur de Lille, U1019-UMR9017-CIIL-Centre d’Infection et d’Immunité de Lille, 59000 Lille, France; 8 Service de Réanimation, Centre Hospitalier de Cayenne, French Guiana, Cayenne, France; 9 Institut für Anästhesiologische Pathophysiologie und Verfahrensentwicklung, Universitätsklinikum Ulm, 89070 Ulm, Germany; 10 Centre AntiPoison de Paris, Hôpital Fernand Widal, APHP, 75010 Paris, France; 11 Faculté de Pharmacie, UMRS 1144, 75006 Paris, France; 12 Université de Paris, UFR de Médecine, 75010 Paris, France; 13 Critical Care Unit, University Hospital of Purpan, Toulouse, France; 14 Toulouse NeuroImaging Center, ToNIC, Inserm 1214, Paul Sabatier University, Toulouse, France; 15 Service de Réanimation Médicale, Groupe Hospitalier de la Région Mulhouse Sud Alsace, Mulhouse, France; 16 INSERM, LNC UMR 1231, FCS Bourgogne Franche Comté LipSTIC LabEx, Dijon, France; 17 Service de Médecine Intensive Réanimation, Sorbonne Université, Hôpitaux Universitaires Pitié Salpêtrière-Charles Foix, Assistance Publique-Hôpitaux de Paris (AP-HP), Paris, France; 18 College de France, Center for Interdisciplinary Research in Biology (CIRB)-UMRS INSERM U1050 - CNRS 7241, Paris, France; 19 Department of Anesthesia and Perioperative Care, University of California, San Francisco, CA 94143 USA; 20 Medical Intensive Care Unit, Saint Antoine University Hospital, APHP, Sorbonne University, 75012 Paris, France; 21 Sorbonne University, Centre de Recherche Saint-Antoine INSERM U938, 75012 Paris, France

**Keywords:** Post-ICU syndrome, Chronic critical illness, Long-term outcome, ICU sequelae, Neuromuscular disorders, Cognitive impairment, Acquired immunosuppression

## Abstract

**Background:**

Post‐intensive care syndrome (PICS) encompasses physical, cognition, and mental impairments persisting after intensive care unit (ICU) discharge. Ultimately it significantly impacts the long‐term prognosis, both in functional outcomes and survival. Thus, survivors often develop permanent disabilities, consume a lot of healthcare resources, and may experience prolonged suffering. This review aims to present the multiple facets of the PICS, decipher its underlying mechanisms, and highlight future research directions.

**Main text:**

This review abridges the translational data underlying the multiple facets of chronic critical illness (CCI) and PICS. We focus first on ICU-acquired weakness, a syndrome characterized by impaired contractility, muscle wasting, and persisting muscle atrophy during the recovery phase, which involves anabolic resistance, impaired capacity of regeneration, mitochondrial dysfunction, and abnormalities in calcium homeostasis. Second, we discuss the clinical relevance of post-ICU cognitive impairment and neuropsychological disability, its association with delirium during the ICU stay, and the putative role of low-grade long-lasting inflammation. Third, we describe the profound and persistent qualitative and quantitative alteration of the innate and adaptive response. Fourth, we discuss the biological mechanisms of the progression from acute to chronic kidney injury, opening the field for renoprotective strategies. Fifth, we report long-lasting pulmonary consequences of ARDS and prolonged mechanical ventilation. Finally, we discuss several specificities in children, including the influence of the child’s pre-ICU condition, development, and maturation.

**Conclusions:**

Recent understandings of the biological substratum of the PICS’ distinct features highlight the need to rethink our patient trajectories in the long term. A better knowledge of this syndrome and precipitating factors is necessary to develop protocols and strategies to alleviate the CCI and PICS and ultimately improve patient recovery.

## Introduction

Critically ill patients’ survivors often acquire multi-organ long-lasting sequelae described as chronic critical illness (CCI) and post-intensive care syndrome (PICS). The CCI is usually defined as a subacute disease state requiring high intensity of care for a protracted period, characterized by lengthy hospital stays, intense suffering, high mortality rates, and substantial resource consumption. In contrast, the PICS represents the remaining health issues caused by the ICU stay after hospital discharge. These terms encompass several distinct pathological and physiological processes that vary among the initial organ injury and the underlying conditions but ultimately impact the functional outcome and long-term survival.

Inflammation, catabolism, and neuroendocrine disorder, also called “Persistent Inflammation, Immunosuppression, and Catabolism Syndrome,” precipitate chronic organ failure and frailty, delay adverse clinical outcomes, and govern clinical trajectories. Therefore, despite a substantial decrease in ICU mortality, the rate of patients discharged to rehabilitation or long-term facilities increases substantially [1]. As today around 50% of ICU patients in OECD countries are over 65 years of age, with increasing comorbidities and frailty criteria, an ICU stay can induce a significant downturn and compromise successful aging.

**Fig. 1:**
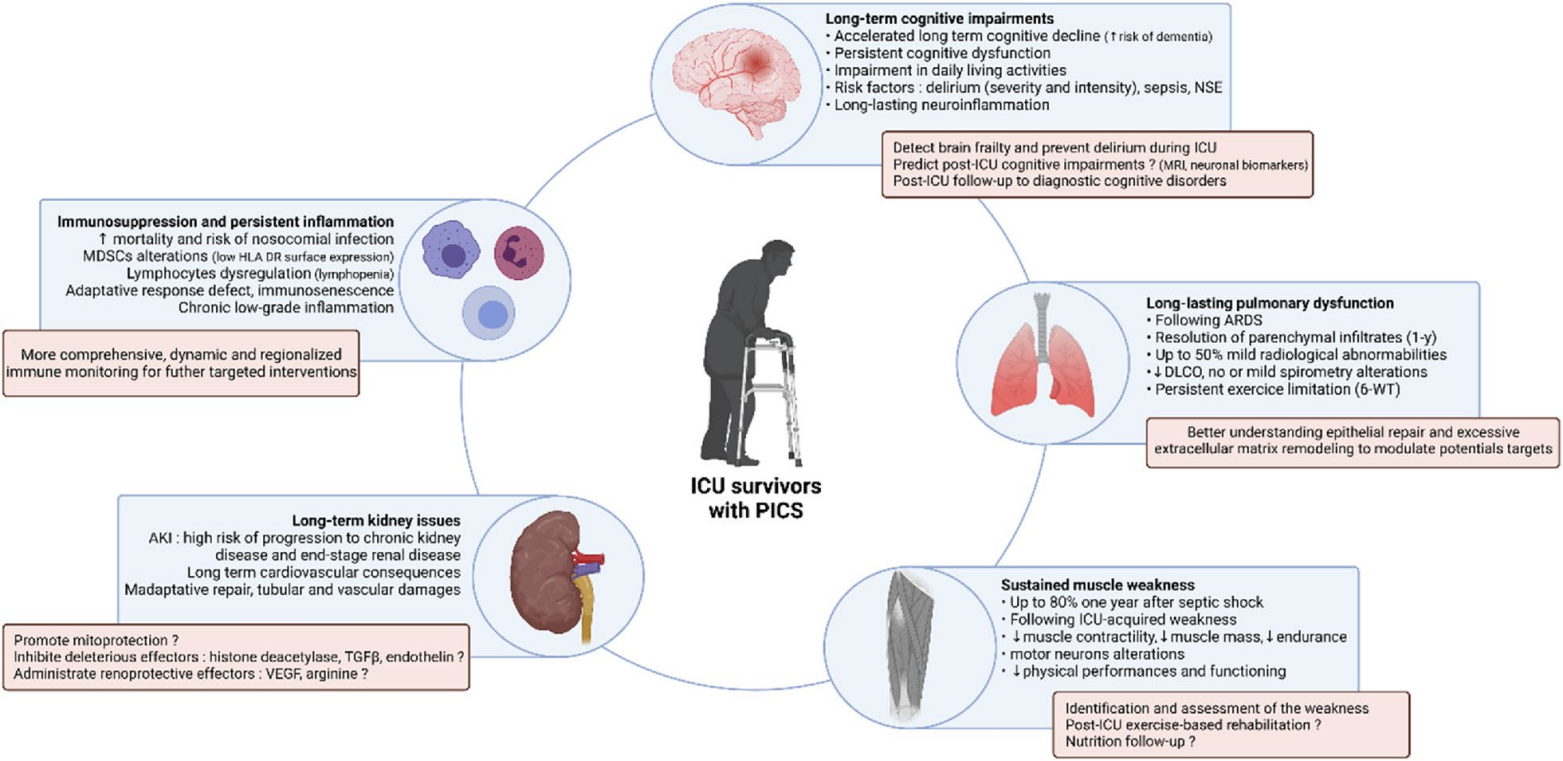
ICU survivors with post-intensive care syndrome. Clinical characteristics and consequences, and futures research directions for each long-lasting sub-syndrome after ICU stay

Today accumulative evidence reported that acute organ failure, even reversible, can cause chronic disorder at a distance or precipitate other systems’ degradation by organs interplays. This review describes the common or organ-specific pathophysiological mechanisms leading to neurocognitive, muscular, respiratory, renal, and cardiovascular long-lasting functional impairment or frailty (Fig. 1). The specific case of children's ICU survivors is also discussed. Each section describes the established or putative cellular mechanisms involved and their potential consequences for the clinician.

### Catabolism syndrome and neuromuscular disorders

Critical illness polyneuropathy (CIP) and critical illness myopathy (CIM), clinically defined by a Medical Research Council (MRC) sum score < 48, contribute unequivocally to ICU-acquired weakness (ICUAW). Patients with ICUAW experience poor short-term consequences (e.g., increased mechanical ventilation duration, length of stay, and in-hospital mortality) [2]. ICUAW also impacts long-term outcomes. A low MRC score at ICU discharge, even below 55, was associated with increased medium and long-term mortality [3–5]. Isolated diaphragmatic dysfunction was not associated with increased 2-year mortality, unlike isolated limb muscle weakness, but when both are combined, the prognosis worsened [5]. Post-ICU follow-up studies also revealed that ICU survivors, including COVID-19 patients [6, 7], frequently exhibited sustained weakness and long-term physical consequences. In the year following ICU discharge, survivors displayed a severe reduction in force development and endurance contraction [8], reported by patients as impacting physical performance and health-related QOL [9]. These consequences persist at 5 years with a persistent limitation in the 6-min walk test [10–12], an impairment in aerobic capacities (decrease in VO2 max) frequently involved in muscle limitations [13], and still have an impact on physical performance and health-related QOL [10–12]. This has been mainly studied after septic shock [9] and ARDS [10–12]. Such consequences on physical performance ultimately impact the autonomy of patients. In one study, almost half of patients who lived at home independently before hospitalization lost this ability 6 months after ICU stay [14]. However, the subsequent trajectory of ICU survivors is variable [15]. While some will improve their functional status, others will never recover. For example, two-thirds of patients surviving septic shock did not recover their previous physical status at 1 year [10]. Diagnostic tools to predict this evolution are currently limited.

Although CIP persists in nearly half of surviving ICU patients [16, 17], both animal and human studies suggest that the neural component is little involved in sustained ICUAW. Conversely, alteration of the muscle component would be the main mechanism [10, 18]. Sustained weakness following CIM is characterized by strength loss due to impaired contractility and muscle wasting**.** Following the acute insult, proteolysis pathways are intensely activated—due to energetic impairment and increased pro-inflammatory mediators—and are directly involved in muscle wasting [19, 20]. Although proteolysis appears to be deleterious to muscle mass, it is essential to muscle homeostasis [21]. In addition, to providing substrates to maintain an energetic level, the ubiquitin–proteasome system (UPS) allows the clearance of non-functional cleaved proteins. In the rat, proteasomal overload was associated with a necrotizing muscle phenotype during long-term critical illness, suggesting an accumulation of proteins insufficiently degraded by UPS [22]. Furthermore, early or late pharmacological inhibition of the proteasome by bortezomib in an experimental model of extensive burn decreased the hypermetabolic muscle response but increased mortality [23].

During the recovery phase, while catabolic pathways are attenuated and protein synthesis is increased, some degree of muscle atrophy persists, suggesting anabolic resistance [24–27]. Animal and human studies have also revealed a decrease in muscle autophagy during the recovery phase of a murine sepsis model [28] and in skeletal muscle of prolonged critically ill patients [29], respectively, impacting muscle contractility. Autophagy is a massive degradation system of damaged cellular components (e.g., mitochondria, damaged organelles, toxic protein aggregates, unfolded or oxidized proteins) that accumulate during critical illnesses due to inflammation and oxidative stress and whose inhibition contributes to various myopathies and muscle wasting [30, 31]. Finally, in response to catabolic muscle damage, the muscle also has a great capacity for regeneration via its satellite cells [32]. This function is also impaired in ICU survivors, with both a decrease in satellite cell content 6 months after ICU stay [24] and aberrant up-regulation of genes involved in the structural and functional muscle development and extracellular matrix remodeling [33], suggesting a defect in the muscle repair process [27]. Moreover, the presence of mitochondrial dysfunction in satellite cells was demonstrated experimentally, and intramuscular injection of mesenchymal cells improved skeletal muscle function and prognosis in mice sepsis [34–36]. Mitochondria play a critical role in muscle physiology and ICUAW pathogenesis [37]. Its dysfunction may contribute to muscle weakness persistence over time. One month after murine sepsis, the mitochondrial population in the skeletal muscle of survivors remained profoundly altered with reduced respiration and severe morphological abnormalities. Mitochondrial dysfunction was also associated with an oxidized protein profile [38]. Abnormalities in calcium homeostasis may also occur due to channelopathies and alter mitochondrial function by calcium overload [39].

Moreover, both animal and human studies have shown persistent muscle weakness despite muscle mass restoration [24, 28, 38], and neuromuscular stimulation of quadriceps in ICU patients restored muscle mass but failed to improve muscle strength in a randomized controlled trial [40]. Overall, these data suggest that the biomechanical quality of muscle fibers seems to be at least as important as their quantity.

The persistence of these deleterious mechanisms which are responsible for sustained ICUAW would be related to persistent inflammation. Despite the insult resolution, a low-grade inflammatory state may persist in ICU survivors. Up-regulation of inflammation genes expressed in the muscle tissue was found at 7 days and 6 months after an ICU stay and was correlated with muscle strength decrease [29]. Persistent inflammation could be a possible explanation for the observed accelerated aging of limb muscles in ICU survivors, a concept described as inflamm-aging [41].

**Fig. 2:**
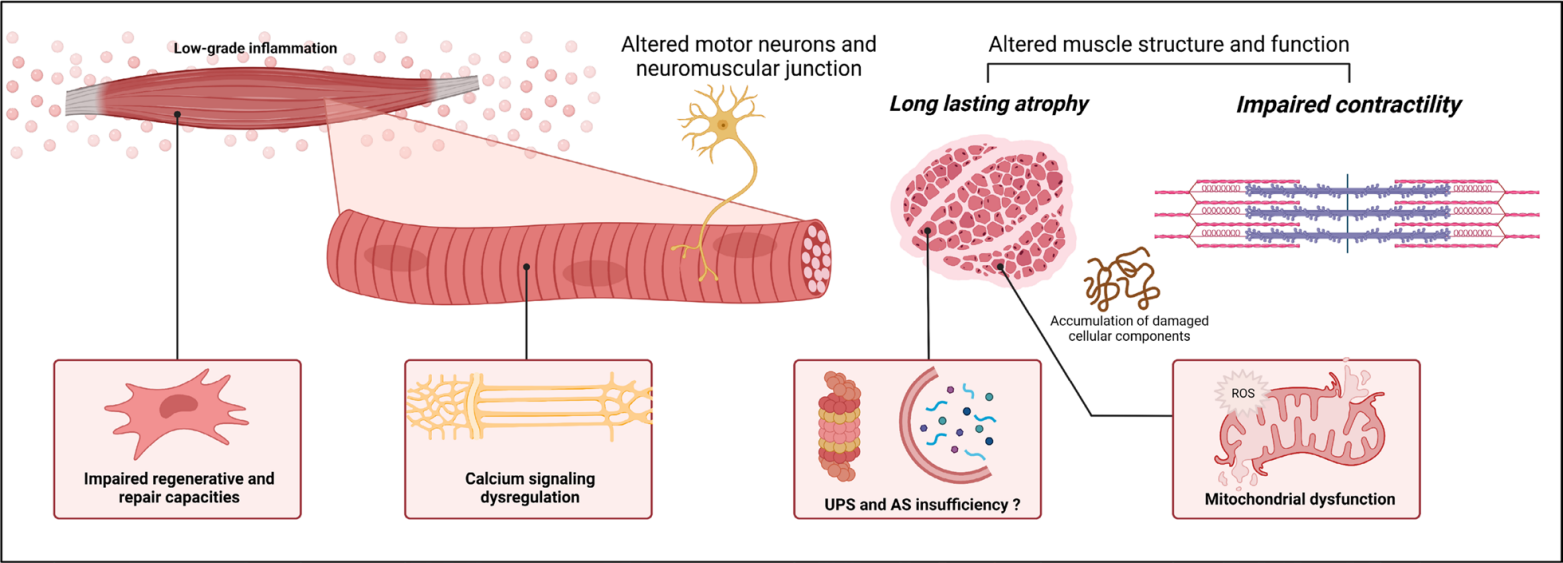
Putative mechanisms of intensive care unit‐acquired weakness

Despite the growing interest in the subject, significant efforts are needed to fill the knowledge gap and better understand the mechanisms underlying accelerated muscle aging in ICU survivors, summarized in Fig. 2. The search for relevant preclinical models to study long-term ICU consequences is crucial to finding innovative therapeutic targets [42]. Currently, there is no pharmacological treatment to prevent or improve weakness after critical illness [43]. Nutritional strategy is of first importance, but its effect could be lessened due to persistent inflammation and anabolism resistance [44]. On top of age-related loss of skeletal muscle mass and function [45], accelerated ICUAW and sarcopenia are a critical issue, particularly in the elderly. At ICU discharge, referral to an appropriate physical rehabilitation route may be valuable but little evidence supports this strategy [46].

### Long-term cognitive impairment and neuropsychic disability

Population-based datasets and prospective cohorts have reported that ICU survivors have a higher odds of a substantial and persistent cognitive downturn and functional disability. One prospective study showed that ICU patients hospitalized for severe sepsis were 3.33 more likely (multivariate analysis) to develop moderate/severe cognitive impairment at one year than to non-septic patients hospitalized in general hospitalization [47]. Similarly, another retrospective study showed that ICU patients hospitalized for sepsis were 2 more likely to develop dementia than age- and sex-matched controls and after adjusting for age, sex, and comorbidities [48]. Using the same methodology but a reverse design, another study revealed that patients with dementia had a higher odds of previous sepsis than the control group. Besides sepsis and septic shock, a prospective study showed that 40% of ICU patients presented global cognition scores that were 1.5 SD below the population means at 3 months post-discharge and that 26% had scored 2 SD below the population mean (equivalent to mild Alzheimer’s disease) whatever the reason for ICU admission [49]. Deficits persisted at 12 months and were responsible for significant impairment in QOL (quality of life) and Instrumental Activities of Daily Living associated with psychopathological symptoms as previously reported in ARDS patients or cardiac surgery [50–52]. Remarkably, in this study, a longer duration of delirium in the hospital was associated with worse global cognition and executive function scores at 3 and 12 months. Since then, this finding has been corroborated in multiple studies, establishing a solid association between delirium severity/duration and long-term cognitive decline acceleration and or severe psychological issues, such as PTSD [53, 54]. In studies focusing on predictive factors of accelerated post-ICU cognitive decline, the intensity of delirium (or need for neuroleptic), sepsis, hypoglycemia, and high NSE seem to be associated with poor cognitive outcomes [55, 56]. Overall, despite some heterogeneity in inclusion criteria, definition and measurement methods/tests for post-ICU cognitive impairment, and long-lasting neuropsychological disorders, there is mounting evidence for the clinical relevance of post-sepsis/post-ICU cognitive impairment and a significant association with delirium/sepsis-associated encephalopathy during the ICU stay [57]. However, the biological substratum of these alterations remains elusive yet.

In a rat model of polymicrobial sepsis (CLP) compared to sham surgery, sepsis directly causes learning and memory impairment even after complete recovery (10 days) [58]. In a non-septic acute inflammation animal model (LPS-induced non-lethal endotoxemia), survivors displayed memory deficits in the radial maze and changes in open field exploratory patterns 3 months after complete recovery [59]. Interestingly these changes were associated with neuroanatomical changes, such as loss of neurons in the hippocampus and the prefrontal cortex and reduced cholinergic innervation in the parietal cortex. Interestingly, in rodent experimental studies, the severity of sepsis (estimated by sepsis behavioral score and plasma IL-6) at 24 h correlated to the increased permeability of the blood–brain barrier in the amygdala, prefrontal cortex, and hippocampus and correlates to persistent oxidative stress in the brain. Ultimately, the sepsis score negatively correlates with cognitive performance in rats at ten days post-CLP [60]. Multiple inflammatory mediators are potentially involved in acute and long-lasting neuroinflammation. Among them, IL-1β seems to play a pivotal role. In one study [61], while WT mice displayed impaired long-term memory consolidation after the LPS challenge, those receiving IL-1 receptor antagonists were protected. It also demonstrated that IL-1β caused hippocampal neuronal dysfunction and may drive neuronal death.

In humans, translational studies are scarce, and most of the literature comes from studies focusing on cognitive decline after major surgery, which is highly relevant in the elderly. In this context, several biomarkers of neuronal injury have been associated with postoperative delirium (POD) and subsequent postoperative cognitive decline (POCD) [62]. Non-specific markers of systemic inflammation, such as CRP, IL-6, IL-1B, or TNF-a, in the blood or CSF are frequently associated with delirium but poorly predict POCD [63–66]. Conversely, biomarkers of neuronal damage, such as S100ß, NSE [67], or phosphorylated neurofilament heavy subunit, are more consistently associated with POD and POCD [68]. In 2012, one study explored ICU survivors in a pilot study combining diffusion tensor imaging MRI, acute delirium monitoring, and cognitive outcomes in 47 patients evaluated after 3 and 12 months of follow-up. They observed that the duration of delirium was associated with significant white matter disruption [defined by a low fractional anisotropy (FA)] at hospital discharge, notably in the corpus callosum and anterior limb of the internal capsule. It also reported that a low FA in the anterior limb of the internal capsule at discharge and in the genu of the corpus callosum at three months was associated with poor cognitive outcomes at 3 and 12 months, suggesting that modern neuroimaging techniques could help screen patients at risk of post-ICU cognitive decline [69].

To date, the mainstream hypothesis is that sepsis/inflammatory critical illness-associated systemic inflammations can induce some acute CNS injury and potentially long-lasting activation of the CNS cells, such as blood–brain barrier endothelial cells [70] or neuroglial cells, and therefore promote a low-grade long-lasting inflammation responsible for neuronal death and neurological disorders. However, experimental data are feeble to support such a statement yet and whether systemic- or SNC-inflammation-induced acute brain dysfunction and long-term neurocognitive disorders occur by overlapping or discrete mechanisms remains to investigate. Nevertheless, because of the strong association with pre-existing mild cognitive impairment and intensity of delirium in the acute setting, measures to detect brain frailty and prevent delirium in ICU should be promoted and belong to a “standard of care bundle in ICU,” with the putative opportunity to limit the long-term cognitive impairment. In addition, older patients and patients at risk might be eligible for a long-term follow-up to favor early diagnosis of mild cognitive impairment or dementia.

### Immunosuppression and persistent inflammation

Following acute critical illness—especially sepsis—a growing body of evidence reveals profound and persistent alteration of immune response in survivors. All cellular components of the immune response studied so far appear to be altered. At the acute phase of critical illness, chemokines, cytokine, and adrenergic storms trigger the rapid mobilization of innate myeloid cells from the spleen and the bone marrow during acute critical illness. This release of functional but mainly immature monocytes and neutrophils, called myeloid-derived suppressive cells (MDSCs), aims at the same time to fight the invading pathogen and initiate the resolution of inflammation. Thus, circulating neutrophils show reduced effector capacity (respiratory burst and chemotaxis), and recent reports reveal that these immature granulocytes (G-MDSCs) are circulating, displaying immunosuppressive properties, with their abundance in the blood being correlated with dire outcomes [71, 72]. Similarly, monocytes display diminished capacity to respond to further insult (experimentally explored by measuring amounts of cytokine production after microbial stimulation). Monocytic MDSC produces a tremendous amount of IL-10 that causes the deactivation of innate and immune cells and has impaired antigen presentation capacities characterized by a low HLA-DR surface expression [73, 74]. Low surface expression of HLA-DR on monocyte membrane is one commonly reported surrogate marker of this immunosuppressive state, strongly correlated with nosocomial infections and mortality. Overall, MDSCs are a heterogeneous population of immature and immunosuppressive myeloid cells, found in critical patients from the early phase to as late as 6 weeks after sepsis onset [75] and are consistently associated with nosocomial infections and bad outcome [76]. Massive MDSC release also creates a void in the bone marrow niche that stimulates the expansion of early myeloid progenitors at the expense of both lymphopoiesis and hematopoiesis [77], which partly explains the persistent lymphopenia and chronic anemia observed in ICU survivors.

**Fig. 3:**
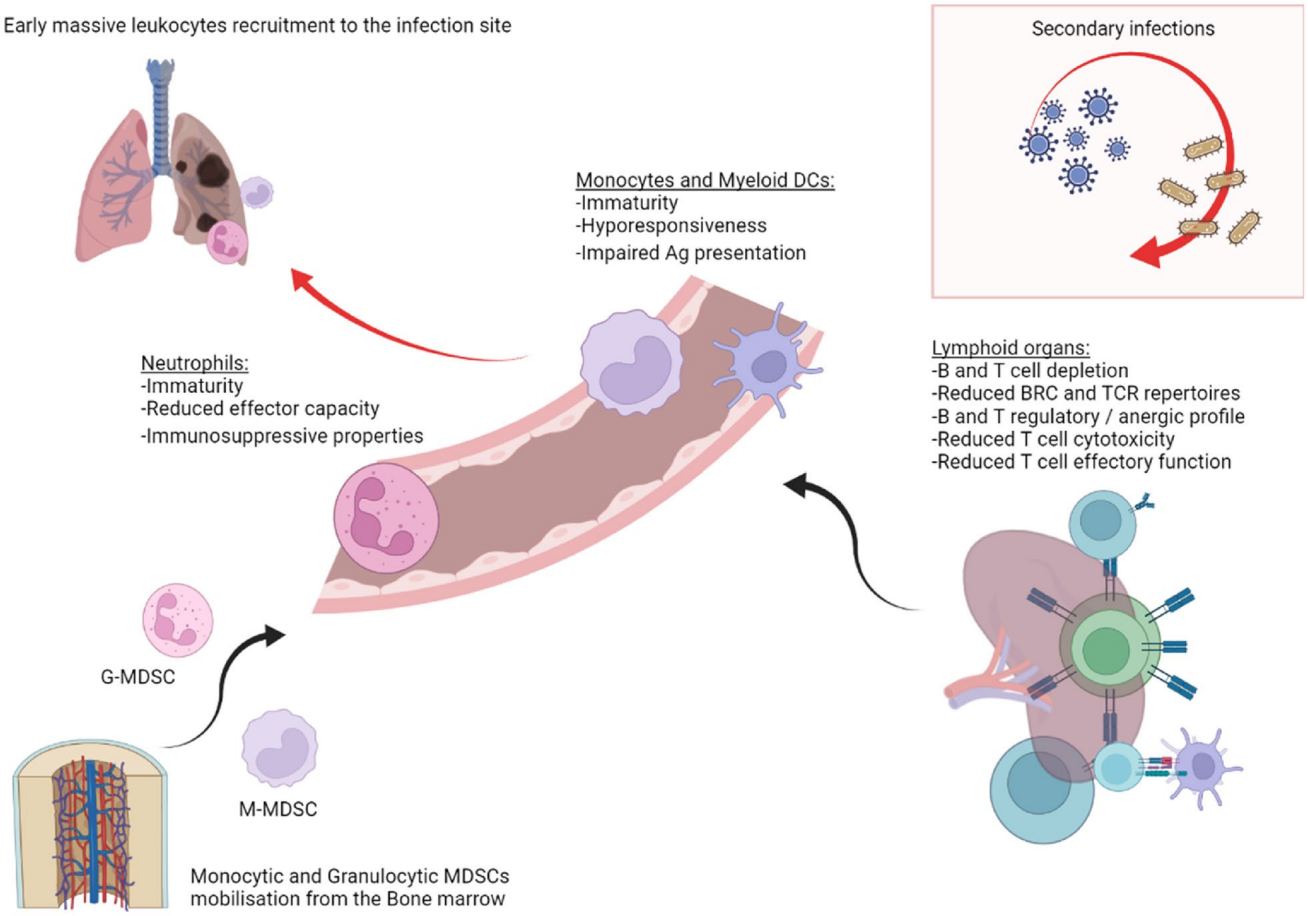
Putative mechanisms of ICU-acquired immune deficiency

Lymphocytes are also involved in immune dysfunction following critical illness. First, lymphopenia is a classical biological feature of sepsis, and persistent lymphopenia on days 3 and 4 after sepsis onset has been associated with nosocomial infection and subsequent death [78]. Data regarding B cells are relatively scarce, but B cell depletion has been documented in early and later phases of critically ill conditions, possibly through increased apoptosis and reduction in B cell maturation [79–81]. Besides B cell depletion, phenotype (B cells subpopulations) and function also seem altered, with data suggesting a switch toward regulatory profile and/or exhausted status, associated with mortality [80, 82]. Regarding T cells, numerous studies have first reported blood depletion during critical illness (especially sepsis) and concomitant apoptosis [83]. Conventional T cells are classically dichotomized as CD4 + or CD8 + , with the CD4 + subset exerting “helper” functions, notably by producing cytokines that shape immune response, and CD8 + being characterized by cytotoxic activities. Different CD4 + T cells subsets are not all similarly affected by apoptosis, and surviving cells undergo multiple phenotypic changes that alter their functions [84]. Thus, the increased proportion of regulatory T cells and decreased proportion of major “helper” subsets (namely, Th1, Th2, and Th17, able to produce IFN) have been documented. Besides numerical changes, functions are also documented, and the main Th subset seems to adopt an “anergic” hyporesponsive state after acute critical illness, characterized by decreased ability to produce cytokine and expression of co-inhibitory receptors. T CD8 + response has also been shown to be altered after experimental sepsis, with decreased response to antigen encounter, reduced proliferative capacity, and ability to clear pathogen [85]. Ultimately, these alterations converge to a defect in the adaptive response, characterized by a massive T and B lymphocytes apoptosis in lymphoid organs, a drastic reduction in their TCR or BCR repertoires, and a decreased effector function leading to insufficient response to further insults, such as nosocomial infections. In addition, persisting low-grade inflammation due to long-lasting tissue injury might perpetuate this phenomenon and paradoxically cause immunosuppression (Fig. 3). In older patients, on top of the immune senescence and inflamm-aging process, the post-septic immune profile notably contributes to the increased incidence and severity of infectious diseases and possibly cancers.

Overall, readers should keep in mind that immune responses are highly dynamic, seldom dichotomic (“pro”/“anti”-inflammatory, for example), and can, at the same time, be compartmentalized. Also, as many human studies on immune function during and after critical illness are conducted in blood, conclusions should not be extrapolated to distant peripheral organs where immune cells exercise their function [86]. Thus, even if we can be affirmative on persistent alteration of immune response after critical illness, a more comprehensive, dynamic—and ideally regionalized—immune monitoring appears a prerequisite for future targeted therapeutic interventions.

### Long-term acute kidney injury issues: risk for progression to chronic kidney disease and systemic consequences

Acute Kidney Injury (AKI) has long been considered an utterly reversible syndrome. However, recent evidence shows that AKI is a major risk factor for progression to Chronic Kidney Disease (CKD) (Hazard Ratio HR 8.8) and end-stage renal disease (HR 3.1) [87], especially in ICU patients [88]; the risk increases with AKI severity. AKI is also associated with cardiovascular risk: congestive heart failure (HR up to 2.2) [87] and acute coronary event (HR 1.7 after renal replacement therapy-requiring AKI) [89]; the risk of death or admission for a major adverse cardiac event is higher after AKI than after myocardial infarction [90]. From those epidemiologic findings emerged the concepts of “maladaptive repair” [91] and interconnection between AKI and CKD [92]. The predominant experimental model for progression from AKI to CKD is ischemia/reperfusion (I/R) in rodents. Two main mechanisms of kidney damage emerge from experimental findings, implying different cell death mechanisms: tubular and vascular damage, both leading to interstitial fibrosis. I/R in proximal tubules induces necroptosis, prolonged expression of pro-inflammatory cytokines (IL-18, IL-1ß and TGF-ß), macrophage infiltrate, and inflammasome activation, with an amplification loop, even after kidney function normalization [93, 94]. Those lesions are responsible for fibrosis and CKD. Mitochondria is a key effector of maladaptive repair. In proximal tubules, ATP dynamic-related protein 1depletion induced by I/R is responsible for mitochondrial fission via Dynamic-Related Protein 1, inducing Reactive Oxygen Species liberation. Therefore, the tubular cell proliferation is inhibited, whereas IL-6 secretion , neutrophils recruitment [95] and apoptosis are increased. Infusion of a mitoprotective agent 1 month after AKI decreases inflammation, restores structural kidney integrity (capillaries and podocytes), and decreases interstitial fibrosis [94]. Autophagy in tubular cells, despite a protective effect on initial AKI, is responsible for more inflammation and worse kidney outcome 30 days after I/R in mice [96], via cell cycle arrest in G2—M phase. It results in up-regulation of profibrotic cytokines (TGF ß, connective tissue growth factor), activation of COL4A1 and COL1A1 genes, and cellular dedifferentiation [97, 98]. The intensity of fibrosis does not depend on the level of apoptosis (preponderant role of cell cycle arrest over apoptosis on fibrosis process). Epigenetic phenomenon is also involved: histone deacetylase inhibition improves long-term kidney function by reducing fibrosis [99]. Peritubular capillary density decreases in the weeks following I/R, despite an initial repair of tubular damage [100]. Thus, sensitivity to angiotensin 2 and hypertension increases. The delayed expression of TGF-ß in ischemic kidney is implied in capillary rarefaction [100, 101], such as endothelin-1, which transcription is sustainably increased after I/R, resulting in a reduction in kidney mass [102]. Capillary rarefaction induces chronic hypoxia, persistent up to 5 weeks after I/R, with elevation in HIF 1 (hypoxia-inducible growth factor) [103]. Basile et al. demonstrated evidence of endothelial–mesenchymal transition, which is much more prevalent than epithelial–mesenchymal transition [104]. Apoptosis also plays a role in capillary rarefaction: indeed, caspase-3 (the main effector of apoptosis) remains activated several weeks after I/R, and caspase-3^−/−^ mice show less microvascular rarefaction and renal fibrosis [103]. In human cell culture, hypoxia enhances apoptosis in endothelial but not epithelial cells, while necrosis is rather a tubular concern [103]. Finally, mitochondrial fission induced by DRP 1 is also implied in capillary rarefaction [95].

**Fig. 4:**
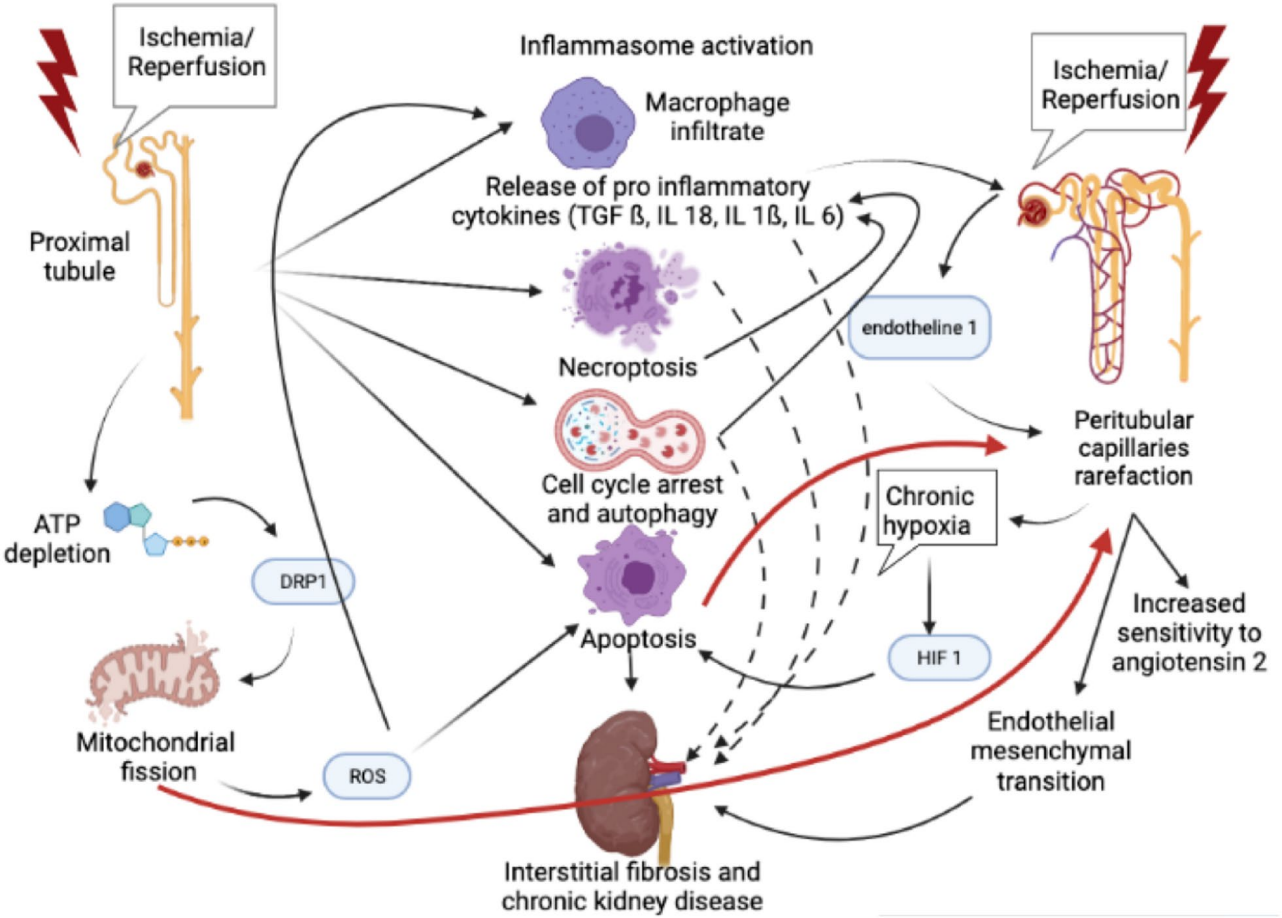
Mechanisms underlying progression toward chronic kidney disease after AKI

Beyond kidney lesions, AKI is a multisystemic concern, with repercussions, for example, in lymph nodes (fibrosis) [105], and lung and brain (increased transcription of pro-inflammatory cytokines) [106, 107]. Among those distant consequences, cardiovascular repercussions are a major concern. I/R in mice induces an increase in TNF-α and IL1, endothelial dysfunction, and cardiomyocytes apoptosis [108] (Fig. 4).

In conclusion, the physiopathology of progression from AKI to CKD has been well described in the last two decades, opening the field for renoprotective interventional studies: mitoprotection [94], inhibition of deleterious effectors, such as histone deacetylase [109], TGF-ß, or endothelin [101], or administration of renoprotective effectors, such as VEGF or arginine [100, 104].

### Long-lasting pulmonary dysfunction after ARDS

The pathogenesis of ARDS involves an extensive insult of distal lung airspaces in response to direct or indirect aggression. Its resolution is an active and complex process that begins from the onset of injury and aims to restore both the structural and functional properties of the lungs. The inflammatory phase resolves through phagocytosis of apoptotic neutrophils by alveolar macrophages, while restoring the alveolar–capillary barrier integrity occurs during the proliferative phase. Epithelial repair involves migration, proliferation, and differentiation of type II alveolar cells associated with fibroblasts influx, aiming to remodel the denuded basement membrane. An inappropriate, extensive, and prolonged inflammation and excessive extracellular matrix deposition and remodeling may lead to residual pulmonary injuries, contributing to the patient’s long-term physical disability. After a sufficient delay, radiological, functional, and physical tests can assess sequelae. Radiological investigations performed after the first year in ARDS survivors may show a complete resolution of parenchymal infiltrates. However, persistent abnormalities, mainly reticular patterns, and ground-glass opacities, are observed in more than half survivors [110–113]. The overall extent is usually low, ranging from 8 to 15% of the lung parenchyma, with a preferential location to the non-dependent regions. Interestingly, patients with ARDS due to primary pulmonary causes, such as pneumonia display more severe fibrotic sequelae than patients with extrapulmonary ARDS [110]. In addition to residual anatomic abnormalities, many ARDS survivors have persistent pulmonary function impairments. The diffusion capacity of the lungs for carbon dioxide (DLCO) improved during the first year post-ARDS, but without reaching the lower limit of the normal range [114]. Thus, one study reported a DLCO of 65% of the predicted value at 6 months, while another described an improvement from 63% at 3 months to 72% at 12 months, then stability over the 4 following years [115–117]. Regarding spirometry, very mild obstructive and restrictive patterns have been described within the first year post-ARDS [114–116, 118]. Forced expiratory volume in one second (FEV_1_) and FEV_1_/forced vital capacity (FVC) ranged, respectively, from 85 to 87% and from 96 to 101% of the predicted value at 12 months, while total lung capacity (TLC) ranged from 88 to 95% of the predicted value at 12 months [114–116, 119]. Interestingly, a protective ventilation strategy (using a low tidal volume) did not provide benefit in terms of long-term pulmonary function in ARDS survivors [114, 118]. Moreover, no difference in lung volumes has been observed between ARDS due to primary pulmonary cause and extrapulmonary ARDS and between patients who had prone positioning or not [110, 119]. Assessment of physical function using the 6-min walking test (6-WT) has shown a significant and persistent exercise limitation in ARDS survivors. At 3 months, one study described a 6MWT distance of 49% of the predicted value, while values ranged from 66 to 72% at 12 months post-ARDS [116, 119]. Notably, this inability to exercise seems disproportionate to mild structural and functional abnormalities reported in ARDS survivors. This discrepancy may be explained by extrapulmonary long-lasting alterations, such as cardiac dysfunction, muscle deconditioning, and neuromyopathy. Accordingly, no correlation has been found between CT scan lesions’ extent or the importance of spirometry abnormalities and the 6MWT distance in ARDS survivors. In conclusion, these findings illustrate long-lasting pulmonary consequences of ARDS, which are relatively mild in terms of radiological abnormalities and spirometry alterations, but more critical in reducing diffusion capacity and exercise limitation.

### Post-intensive care syndrome in children

Like adults, child PICU survivors may develop deterioration in physical, cognitive, social, and psychological functioning [120]. Recently, the conceptualization of PICS in children has led to the development of the PICS-Pediatrics framework. Factors specific to child health are critical to note. Critical illnesses occur at a time of tremendous growth and maturation, and an increasing proportion of children admitted to intensive care units have chronic diseases and developmental disorders at baseline. Consistently, upon discharge, the child's physical, cognitive, emotional, and social health are strongly influenced by the child’s pre-PICU condition, development, and maturation. Family, parents, and siblings’ emotional and social health may also be affected. Therefore, the trajectory and duration of recovery are highly variable [121].

#### Physical function impairment

PICU-acquired new dysfunctions (respiratory disabilities, pain, poor mobility, and impaired self-care and feeding) occur in 10% of all admissions [122, 123]. A Functional Status Scale (FSS), involving six domains (mental status, sensory, communication, motor function, feeding, and breathing), has been recently developed [124]. Identified risks for poor functional outcomes include baseline disability, admission for trauma, neurologic or oncologic disorders, cardiac arrest, age < 1 year, and disease severity [120, 125]. Children with normal baseline function experience a more significant functional decline, albeit with faster recovery than those with impaired baseline function [126]. The FSS does not assess sleep disturbance, fatigue, and severe weakness which may be underestimated [127].

#### Pediatric ICU-acquired weakness (PICUAW)

Unlike adults, data on PICUAW are limited, but it reportedly occurs in 1.7–4.7% of PICU survivors, which is much lower than that in adults [128, 129]. This discrepancy remains unclear, but differences between children and adults can be considered: children’s axons are shorter making them less susceptible to injury, and children have better mitochondrial function and higher/better restorative neurotrophic factor concentration/function. Consistently, they recover better from immune-mediated peripheral neuropathies [130]. Furthermore, children have fewer pre-existing nerve or muscle-damaging medical conditions, like diabetes, cancer, chronic organ failure, or chronic drug use. However, studies focusing on high-risk groups with multi-organ failure, severe sepsis, patients with high-frequency oscillatory ventilation, ARDS, or polytrauma, may better estimate the real risk of PICUAW in susceptible critically ill children.

#### Lung consequences of prolonged mechanical ventilation in children

Experimental pediatric data show an age-related susceptibility to VILI [131]. The concentration of elastin in the infant’s lung increases tenfold during the first 20 days of life and then increases less rapidly. Collagen concentration increases linearly from infancy to childhood [132]. Differences in lung elastic properties may account for differences in lung strain. Age-dependent differences in NF-κB have been described in animal models, showing less inflammation in neonatal mice after exposure to hyperoxia [133]. Injurious mechanical ventilation does not activate innate immunity in infants or young children to the same extent as in adults, because the full capacity of the innate immune system is not reached until adolescence [134]. Adaptive immunity also differs between young children and adults, with a tendency toward a more anti-inflammatory response in children [135]. In summary, pediatric patients may be less susceptible than adults to VILI [136].

#### Pediatric neurocritical illness and cognitive function impairment

To assess the onset of new cognitive impairment, the 6-point Pediatric Brain Performance Category (PBPC) is commonly used to estimate baseline global cognitive function and changes during and after the PICU stay [137]. Cognitive decline reportedly occurs in 3.4% of PICU survivors. Risk factors include admission diagnosis of trauma, poisoning, neurologic disease or cancer, invasive mechanical ventilation, and extracorporeal life support [138]. Acute neurological condition was the most significant predictor of an adverse cognitive outcome at 6 months, after adjusting for illness severity and pre-PICU functioning [139]. Blood biomarkers of injury, inflammation, regeneration, and plasticity may be useful in assessing the risk of functional impairment after acute brain injury [140]. In children with traumatic brain injury or cardiac arrest, diagnostic and prognostic biomarkers, such as NSE and S100b, have been evaluated [141, 142]. Recently, low blood levels of brain-derived neurotrophic factor and vascular endothelial growth factor, which are biomarkers of regeneration, have identified children at risk for new cognitive impairment among survivors of pediatric neurocritical care [143]. Ultimately, the cognitive impairments will evolve afterward and the PCPC score at PICU discharge may worsen or improve [120].

#### Psychological function impairment

From 17 to 62% of PICU survivors experienced post-traumatic stress disorder (PTSD) [121, 144]. Depression, changes in self-esteem, delusional memories or fears, and sleep disturbances have also been reported in children after PICU discharge. Pieces of evidence suggest that children with psychiatric morbidity after discharge are more likely to be readmitted for physical problems in the following 6–12 months [145]. Several factors increase the risk of psychological issues. Delusional memories were independently associated with length of sedation and subsequent PTSD symptoms when adjusting for illness severity and emergency admission status. Conversely, factual memories of the ICU stay were not associated with PTSD symptoms, but emergency admission status was, as were illness severity, exposure to invasive procedures, and sepsis [146].

#### Social manifestations and PICS family

Qualitative studies of PICU survivors have revealed themes related to disrupted lives, social stigma, and the need to rebuild social identities, particularly in older children [147]. Parents of PICU survivors may experience post-traumatic stress, anxiety, and depression symptoms shortly after discharge and during the recovery process [148]. Risk factors for long-term problems include unexpected PICU admission and the number of medical procedures performed in the PICU, as well as a history of traumatic events, psychological problems before PICU admission, limited social support, and negative memories of the PICU stay [109, 120].

### Conclusion

Despite significant improvement in global ICU survival, patients often develop a multi-faceted post-ICU syndrome, encompassing multi-organ frailty and causing substantial impairment in QOL. Given that ICU's population is rapidly aging in western countries, these issues are particularly meaningful. As a research agenda, a better understanding of the underlying biological mechanisms linking acute disorder and long-lasting impairment through translational studies is a matter of great priority, such as clinical trials to explore interventions to prevent or treat PICS. Nevertheless, this field of research is challenging due to the multiple facets of PICS, and the need for long-term follow-up collaborative studies.

Therefore, we believe that general practitioners, internists, or geriatricians must be sensitized alongside with intensivists to long-term follow-up of ICU survivors and its specific issues, so that patients could benefit durably and thoroughly from the in-ICU mortality reduction observed over the last 20 years.

## Data Availability

Not applicable.

## Abbreviations

6MWT: 6 minutes’ walk test
AKI: Acute kidney injury
ARDS: Acute respiratory distress syndrome
BCR: B cell receptors
CCI: Chronic critical illness
CD: Cluster of differentiation
CIM: Critical illness myopathy
CIP: Critical illness polyneuropathy
CKD: Chronic kidney disease
CLP: Cecal ligation and puncture
CNS: Central nervous system
CRP: C-reactive procedure
DLCO: Diffusion capacity of the lungs for carbon dioxide
EMG: Electromyography
FA: Fractional anisotropy
FEV: Forced expiratory volume
FSS: Functional status scale
HR: Hazard ratio
I/R: Ischemia/reperfusion
ICU: Intensive care unit
ICUAW: ICU-acquired weakness
IFN: Interferon
IL: Interleukin
LTCI: Long-term cognitive impairment
MDSC: Myeloid-derived suppressive cells
MRC: Medical Research Council
NSE: Neuron-specific enolase
PCPC: Pediatric cerebral performance category
PICS: Post‐intensive care syndrome
POCD: Postoperative cognitive decline
POD: Postoperative delirium
PTSD: Post-traumatic stress disorder
QOL: Quality of life
SF36: Short Form-36
TCR: T cell receptors
TLC: Total lung capacity
TNF: Tumor necrosis factor
UPS: Ubiquitin–proteasome system
VEGF: Vascular endothelial growth factor
VILI: Ventilator-induced lung injury
